# Leptin OB3 peptide suppresses leptin-induced signaling and progression in ovarian cancer cells

**DOI:** 10.1186/s12929-017-0356-6

**Published:** 2017-07-27

**Authors:** Yu-Tang Chin, Le-Ming Wang, Meng-Ti Hsieh, Ya-Jung Shih, André Wendindondé Nana, Chun A. Changou, Yu-Chen S. H. Yang, Hsien-Chung Chiu, Earl Fu, Paul J. Davis, Heng-Yuan Tang, Hung-Yun Lin

**Affiliations:** 1 Taipei Cancer Center, Taipei Medical University, Taipei, Taiwan; 2 Department of Dentistry, Wan Fang Hospital, Taipei Medical University, Taipei, Taiwan; 3 Department of Obstetrics and Gynecology, Wan-Fang Hospital, Taipei, Taiwan; 4 PhD Program for Cancer Biology and Drug Discovery, College of Medical Science and Technology, Taipei Medical University, 250 Wu-Hsing Street, Taipei, 11031 Taiwan; 5 Integrated Laboratory, Center of Translational Medicine, Taipei Medical University, Taipei, Taiwan; 6 Core Facility, Taipei Medical University, Taipei, Taiwan; 7 Joint Biobank, Office of Human Research, Taipei Medical University, Taipei, Taiwan; 8 Department of Periodontology, School of Dentistry, National Defense Medical Center and Tri-Service General Hospital, Taipei, Taiwan; 9 Department of Dentistry, Taipei Tzu Chi Hospital Buddhist Tzu Chi Medical Foundation, New Taipei City, Taiwan; 10 Pharmaceutical Research Institute, Albany College of Pharmacy and Health Sciences, Albany, NY USA

**Keywords:** Obesity, Leptin, OB3-leptin peptide, Ovarian cancer

## Abstract

**Background:**

Obesity and its comorbidities constitute a serious health burden worldwide. Leptin plays an important role in diet control; however, it has a stimulatory potential on cancer cell proliferation. The OB3 peptide, a synthetic peptide, was shown to be more active than leptin in regulating metabolism but with no mitogenic effects in cancer cells.

**Methods:**

In this study, we investigated the proliferative effects, gene expressions and signaling pathways modulated by leptin and OB3 in human ovarian cancer cells. In addition, an animal study was performed.

**Results:**

Leptin, but not OB3, induced the proliferation of ovarian cancer cells. Interestingly, OB3 blocked the leptin-induced proliferative effect when it was co-applied with leptin. Both leptin and OB3 activated the phosphatidylinositol-3-kinase (PI3K) signal transduction pathway. In addition, leptin stimulated the phosphorylation of signal transducer and activator of transcription-3 (STAT3) Tyr-705 as well as estrogen receptor (ER)α, and the expression of ERα-responsive genes. Interestingly, all leptin-induced signal activation and gene expressions were blocked by the co-incubation with OB3 and the inhibition of extracellular signal-regulated kinase (ERK)1/2. Coincidently, leptin, but not OB3, increased circulating levels of follicle-stimulating hormone (FSH) which is known to play important roles in the initiation and proliferation of ovarian cancer cells.

**Conclusions:**

In summary, our findings suggest that the OB3 peptide may prevent leptin-induced ovarian cancer initiation and progression by disrupting leptin-induced proliferative signals via STAT3 phosphorylation and ERα activation. Therefore, the OB3 peptide is a potential anticancer agent that might be employed to prevent leptin-induced cancers in obese people.

## Background

Obesity is a big concern in modern society and controlling the diet is acknowledged as very important. Although long-term solution lies in lifestyle changes in terms of dieting and exercise, drugs, medicinal foods, and dietary supplements are required for those who are already obese. However, the mitogenic effects of those drugs and dietary supplements have not been fully investigated.

A high-fat diet associated with obesity may alter multiple molecular factors that act synergistically to increase the risk of colon cancer associated with obesity [1]. Evidences showed that obesity is related to several types of cancers [2]. The obesity-related hormone, leptin, secreted by adipose tissues, has a close relationship with the hypothalamo-pituitary-thyroid axis [3]. Leptin stimulates thyrotropin-releasing hormone (TRH) expression in the hypothalamus, and subsequent production of the thyroid-stimulating hormone (TSH) [4]. Leptin is mitogenic in various cell types, including hematopoietic cells, normal and transformed epithelial cells and vascular endothelial cells [5, 6]. It is considered to be involved in the onset and progression of several types of cancers including colorectal, breast, endometrial, and esophageal cancers. Serum leptin is present in patients with breast cancer, and concentrations are higher in women with high-grade tumors [7]. A body-mass index (BMI) ≥30 kg/m^2^ is correlated with increased expression of leptin receptors (OB-Rs) [8]. Both long- (OB-RB) and short-form (OB-RA) leptin receptors are expressed in normal and neoplastic ovarian tissues. OB-R expression observed in ovarian cancer cells is associated with tumor aggressiveness [9] and a higher incidence of lymph node metastasis [10]. Furthermore, OB-R expression in neoplastic tissues is around 5-fold higher than that in normal tissues, and a role of OB-RB in endometrial carcinogenesis was proposed [8]. Evidence showed that leptin may play a role in the development of ovarian cancers [11, 12]; however, its prognostic value is still undetermined, and the molecular basis of these effects also remains unclear.

Leptin stimulates proliferation of breast cancer [13], hepatoma cells [14] and prostate cancer [15, 16]. However, our recent studies indicated that leptin does not stimulate the proliferation of different thyroid cancer cells [17]. In addition, leptin acting via the OB-R may regulate the migration of cancer cells [9]. In anaplastic thyroid cancer cells, leptin stimulated cell invasion, but reduced adhesion [17].

The elevated leptin-led promotion of cancer progression is associated with the Janus kinase 2 (JAK)-signaling transducer and activator of transcription (STAT)-3 signaling pathway and STAT3 target gene expression [18, 19]. Recruitment via the Src homology 2 domain to receptor phosphotyrosine peptide motifs facilitates STAT phosphorylation on a key tyrosyl residue by growth factor receptors and JAK [20]. Activated phosphorylation induces STAT-STAT dimerization and nuclear translocation and eventual binding to specific DNA-response elements in the promoters of target genes that activates gene transcription [21].

A new class of functional leptin peptides was synthesized [22–24]. Effects of the leptin-related synthetic peptide, OB3, on energy balance and glucose homeostasis in ob/ob and db/db mice were confirmed [24, 25]. More importantly, it did not stimulate cell proliferation at least in human cervical cancer HeLa cells or thyroid cancer cells [17]. Studies also indicated that the OB3 peptide induces STAT3 phosphorylation by extracellular signal-regulated kinas (ERK)1/2 and phosphatidylinositol-3-kinase (PI3K)-dependent signal transduction [22]. Phosphorylation of Tyr-705 of STAT3 is induced by leptin and OB3 in anaplastic thyroid cancer cells. On the other hand, neither OB3 nor leptin activated the ERK1/2 or PI3K signal transduction pathways in follicular or papillary thyroid cancer cells [17].

In the present study, we compared and analyzed signal transduction and mechanisms which were activated by leptin and the OB3 peptide in ovarian cancer cells. We found that Leptin, but not OB3 peptide, stimulated ovarian cancer cell proliferation and gene expression. Additionally, activated ERK1/2 was essential for the phosphorylation of STAT3 Tyr-705 and ERα, and expressions of ERα-responsive genes. All of these leptin-induced activities including proliferation were suppressed by co-application of the OB3 peptide. Finally, evidence proved that leptin, but not OB3, increased blood serum follicle-stimulating hormone (FSH) which was proven to play important roles in the initiation and proliferation of breast cancer and gynecological cancers.

## Methods

### Cell lines

Human ovarian cancer SKOV-3 (ATCC® HTB-77™) and OVCAR-3 (ATCC® HTB-161™) cells were purchased from American Type Culture Collection (ATCC, Manassas, VA, USA) by the Bioresource Collection and Research Center (BCRC, Hsinchu, Taiwan). These cell lines were tested and authenticated by the BCRC. We purchased them from BCRC and passaged them for less than 6 months after thawing and maintained them for further study in RPMI 1640 medium supplemented with 10% fetal bovine serum (FBS) (for SKOV-3 cells) or with 20% FBS/0.01 mg/ml bovine insulin (for OVCAR-3 cells). All cell cultures were maintained in a 5% CO_2_/95% air incubator at 37 °*C. prior* to treatment, cells were placed in 0.25% hormone-stripped FBS-containing medium for 2 days.

#### OB3 and leptin peptide

Human and mouse leptin peptides were purchased from Sigma-Aldrich (St. Louis, MO, USA). OB3 peptides (human OB3 peptide amino acid sequence: SCHLPWA; and mouse OB3 peptide amino acid sequence: SCSLPQT) were synthesized and confirmed as described previously [26, 27].

#### Cell viability assay

SKOV-3 cells (5000 cells per well) and OVCAR-3 cells (10^4^ cells per well) were seeded in 6-well plates and treated with different concentrations of leptin (1, 10, and 100 nM), OB3 (1, 10, and 100 μM) or combined treatment (10 nM leptin plus OB3 1, 10, and 100 nM; 100 nM leptin plus OB3 1, 10, and 100 μM) for 4 days with re-flashed medium and peptides daily. Cell proliferation was determined by counting the number of cells with a Countess™ Automated Cell Counter (Thermo Fisher Scientific, Waltham, MA, USA). Triplicate wells were assayed for each experiment and three independent experiments were performed. Data are expressed as the mean of cell number ± standard deviation (SD).

#### Western blot analysis

To examine the effects of OB3 on leptin-induced proliferative proteins and signaling pathways, we performed a Western blot analysis to quantify protein expression levels of cyclin D1, proliferating cell nuclear antigen (PCNA), pSTAT3(Tyr705), pPI3K(p85), pERK1/2, pERα and ERα in total cell lysates of SKOV-3 cells which were treated with 10 nM leptin, OB3 (10 μM OB3 for examining signaling pathways; 100 μM OB3 for examining proliferative proteins) and their combination. Protein samples were resolved on 10% sodium dodecyl sulfate polyacrylamide gel electrophoresis (SDS-PAGE). A 40-μg quantity of protein was loaded in each well with 5× sample buffer, and protein samples were resolved by electrophoresis at 100 V for 2 h. The resolved proteins were transferred from the polyacrylamide gel to Millipore Immobilon-PSQ Transfer nitrocellulose membranes (Millipore, Billerica, MA, USA) with the Mini Trans-Blot® Cell (Bio-Rad Laboratories, Hercules, CA, USA). Membranes were blocked with a solution of 2% bovine serum albumin (BSA) in Tris-buffered saline. Membranes were incubated with primary antibodies to cyclin D1, phosphor-STAT3(Tyr705), pPI3K(p85), phospho-p44/42 mitogen-activated protein kinase (MAPK) (pERK1/2), phospho-ERα (Ser167) (pERα), ERα (Cell Signaling Technology, Beverly, MA, USA), PCNA, and GAPDH (GeneTex International, Hsinchu City, Taiwan), at 4 °C overnight and washed, and the proteins were detected with horseradish peroxidase (HRP)-conjugated secondary antibodies and Immobilon™ Western HRP Substrate Luminol Reagent (Millipore). Images of the Western blots were visualized and recorded by Amersham Imager 600 (GE Healthcare Life Sciences, Pittsburgh, PA, USA).

#### Transfection of short hairpin (sh)RNA

To evaluate the role of the leptin receptor (OB-R) in OB3-suppressed leptin-induced cell proliferation, the shRNA of OB-R was used to knockdown the expression of OB-R. SKOV-3 cells were seeded onto 6-well tissue culture plates at 80%–90% confluence (10^5^ cells /well), and maintained in the absence of antibiotics for 24 h before transfection. The culture medium was removed prior to transfection, and cells were washed once with phosphate-buffered saline (PBS) then transfected with an OB-R shRNA expression plasmid (TRCN0000058801) or a scrambled plasmid (ASN0000000004) (0.2 μg/well, RNAi Core Facility, Academia Sinica, Taipei, Taiwan) using Lipofectamine 2000 (2 μg/well) in Opti-MEM I medium according to instructions of the manufacturer (Thermo Fisher Scientific, Waltham, MA, USA). After transfection, cultures were incubated at 37 °C for 6 h and then placed in fresh culture medium. After an additional 48 h, cells were studied.

#### Quantitative real-time polymerase chain reaction (qPCR)

To examine the effects of leptin and OB3 on mRNA expression of proliferative, metastatic and ERα-responsive genes, we treated SKOV-3 cells with vehicle, leptin (1, 10, and 100 nM), OB3 (1, 10, and 100 μM), and their combination (10 nM leptin plus OB3 1, 10, and 100 nM) for 12 h. In addition, messenger (m)RNA expressions of OB-R in two human ovarian cancer cells (SKOV-3 and OVCAR-3) and the effect of leptin or OB3 on OB-R-knockdown in SKOV-3 cells were also investigated. Total RNA was extracted and genomic DNA was also eliminated with an illustra RNAspin Mini RNA Isolation Kit (GE Healthcare Life Sciences, Buckinghamshire, UK). DNase I-treated total RNA at 1 μg was reverse-transcribed with a RevertAid H Minus First Strand cDNA Synthesis Kit (Life Technologies, Carlsbad, CA, USA) into complementary (c)DNA, and used as the template for real-time PCR and analysis. The real-time PCRs were performed using a QuantiNova™ SYBR® Green PCR Kit (Qiagen, Hilden, Germany) on a CFX Connect™ Real-Time PCR Detection System (Bio-Rad Laboratories, Hercules, CA, USA). This involved an initial denaturation at 95 °C for 5 min, followed by 45 cycles of denaturing at 95 °C for 5 s and combined annealing/extension at 60 °C for 10 s, as described in the manufacturer’s instructions. Primer sequences were as followed: *Homo sapiens* cyclin D1 (*CCND1*), forward 5′-CAAGGCCTGAACCTGAGGAG-3′ and reverse 5′-GATCACTCTGGAGAGGAAGCG-3′ (accession no.: NM_053056); *Homo sapiens PCNA*, forward 5′-TCTGAGGGCTTCGACACCTA-3′ and reverse 5′-TCATTGCCGGCGCATTTTAG-3′ (accession no.: BC062439.1); *Homo sapiens* v-myc avian myelocytomatosis viral oncogene homolog, (*c-Myc*), forward 5′-TTCGGGTAGTGGAAAACCAG-3′ and reverse 5′-CAGCAGCTCGAATTTCTTCC-3′ (accession no.: NM_002467); *Homo sapiens* matrix metalloproteinase 2 (*MMP2*), forward 5′-ATCCAGACTTCCTCAGGCGG-3′ and reverse 5′-CCTGGCAATCCCTTTGTATGTT-3′ (accession no.: NM_004530.5); *Homo sapiens* matrix metalloproteinase 9 (*MMP9*), forward 5′-TGTACCGCTATGGTTACACTCG-3′ and reverse 5′-GGCAGGGACAGTTGCTTCT-3′ (accession no.: NM_004994.2); *Homo sapiens* hypoxia-inducible factor 1, alpha subunit (*HIF-1α*), forward 5′-TGAACGTCGAAAAGAAAAGTCTCG-3′ and reverse 5′-GGAAGTGGCAACTGATGAGC-3′ (accession no.: NM_001243084.1); *Homo sapiens* vascular endothelial growth factor A (*VEGF-A*), forward 5′-TACCTCCACCATGCCAAGTG-3′ and reverse 5′-GATGATTCTGCCCTCCTCCTT-3′ (accession no.: NM_001204384.1); *Homo sapiens* estrogen receptor 1 (*ERα*), forward 5′-TCTTGGACAGGAACCAGGGA-3′ and reverse 5′-TGATGTAGCCAGCAGCATGT-3′ (accession no.: NM_000125.3); *Homo sapiens* leptin receptor (*OB-R*), forward 5′-GTGGGGCTATTGGACTGACT-3′ and reverse 5′-TTCAGAGAAGTACACCCATAATCCT-3′ (accession no.: NM_002303); and *Homo sapiens* 18S ribosomal RNA (*18S*), forward 5′-GTAACCCGTTGAACCCCATT-3′ and reverse 5′-CCATCCAATCGGTAGTAGCG-3′ (accession no. NR_003286). Calculations of relative gene expressions (normalized to the 18 s reference gene) were performed according to the ΔΔCT method. The fidelity of the PCR was determined by a melting point analysis.

#### Animal study

Nude mice (BALB/cAnN.Cg-Foxn1nu/CrlNarl) were purchased from the National Laboratory Animal Center (Taipei, Taiwan), were housed in a reserved, pathogen-free facility and were handled in accordance with protocols approved by the Institutional Animal Care and Use Committee of the National Defense Medical Center, Taipei, Taiwan (IACUC-15-340). To determine whether leptin or OB3 affected circulating levels of LH or FSH, we intraperitoneally injected mice with 80 μg/kg mouse leptin or 1 mg/kg mouse OB3. Ten-week-old male nude mice (*n* = 15), weighing 20 ~ 25 g, were randomly divided into three treatment groups, defined as follows: a control group (saline injection, *n* = 5), a leptin group (80 μg/kg leptin injection, *n* = 5), and an OB3 group (1 mg/kg OB3, *n* = 5). Blood samples were collected on the 7th day before injection and the 2nd day after injection. Serum samples were separated by centrifugation and stored at −80 °C.

#### Detection of FSH and LH by a competitive enzyme-linked immunosorbent assay (ELISA)

Levels of FSH and LH in aliquots of serum were measured by competitive ELISA with commercial test kits: a Mouse FSH ELISA Kit (catalog no.:MBS2507988, MyBioSource, San Diego, CA, USA) and a LH ELISA Kit (catalog no.: ABIN415551, Antibodies-online, Aachen, Germany). All ELISA examinations were carried out according to the manufacturer’s instructions.

#### Data analysis and statistics

Western blotting densities were measured and gene expression of the real-time qPCR was analyzed by IBM^→^ SPSS^→^ Statistics software vers. 19.0 (SPSS, Chicago, IL, USA). Student’s *t*-test was conducted and considered significant at *p*- values of <0.05, (*, ^#^, ^$^), 0.01 (**, ^##^, ^$$^) and 0.001 (***, ^###^, ^$$$^), with power 0.80.

## Results

### Leptin, but not the OB3 peptide, stimulates cell proliferation and gene expression in ovarian cancer cells

**Fig. 1:**
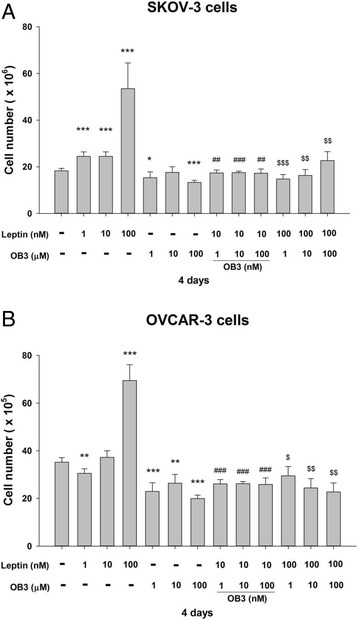
Effects of leptin and the OB3 peptide on ovarian cancer cell proliferation. **a** SKOV-3 and **(b)** OVCAR-3 cells were seeded in 6-well plates and treated with different concentrations of the leptin peptide, OB3 peptide, or their combination as indicated, with re-flashed medium daily for 4 days. Cell proliferation was examined by cell counting. Data are the mean ± SD of separate independent experiments, *n* = 5. (* *p* < 0.05, ** *p* < 0.01, *** *p* < 0.001 compared to the control group (non-treatment); ^##^ *p* < 0.01, ^###^ *p* < 0.001 compared to 10 nM leptin; ^$^ *p* < 0.05, ^$$^ *p* < 0.01, ^$$$^ *p* < 0.001 were compared to 100 nM leptin)

Leptin and its receptor (OB-R) were shown to stimulate the proliferation of ovarian cancer cells [28, 29]. Studies were conducted on the effects of leptin and the OB3 peptide on cell proliferation in ovarian cancer cells. Ovarian cancer SKOV-3 and OVCAR-3 cells were treated with different concentrations of leptin (1 ~ 100 nM) or OB3 (1 ~ 100 μM) for 4 days with re-flashed medium and peptides daily. Results from cell counts showed that treatment of SKOV-3 cells with leptin stimulated cell proliferation in a concentration-dependent manner (Fig. 1a), but treatment with OB3 did not induce changes in cell proliferation (Fig. 1a), even though the concentration was 100-fold higher than that of leptin. Additionally, 100 μM of OB3 significantly inhibited SKOV-3 cell proliferation (27.1%). These results agreed with a previous report that the leptin peptide has a proliferative effect on ovarian cancer cells [12]. Interestingly, the proliferative effect of leptin was inhibited by co-incubation with OB3 (Fig. 1a) even though the concentration of OB3 was as low as 1 μM (39.8%).

Parallel studies were conducted in another ovarian cancer cell line, OVCAR-3 cells. Leptin stimulated cell proliferation, but OB3 did not (Fig. 1b). Leptin-induced proliferation was inhibited by OB3 (Fig. 1b). OB3 (1 ~ 100 μM) had almost the same inhibitory effect on 10 nM of leptin-induced cell proliferation (Fig. 1b). Interestingly, the inhibitory effect on 100 nM of leptin-induced cell proliferation induced by OB3 was inversely proportional to the concentration of OB3 (1 ~ 100 μM; 57.6%, 64.8% ~ 67.3%) (Fig. 1b).

**Fig. 2:**
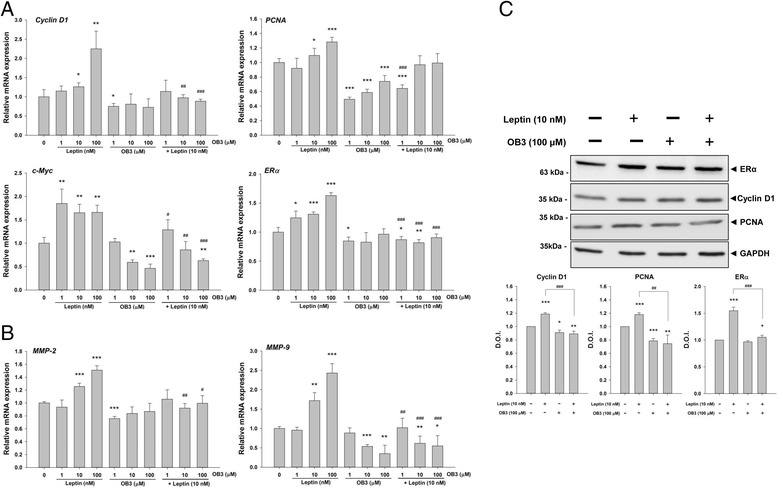
Effects of leptin and the OB3 peptide on expressions of proliferative and metastasis-related genes in ovarian cancer cells. SKOV-3 cells were seeded in 6-well plates and treated with different concentrations of the leptin peptide, OB3 peptide, or their combination for 8 or 24 h. Cells were harvested and total RNA or proteins were extracted. qPCR experiments were conducted to examine expression of proliferative genes (*Cyclin D1, PCNA*, *c-Myc* and *ERα*) **(a)** and metastasis-related genes (*MMP-2* and *MMP-9*) **(b)**. Western blotting experiments were performed to investigate the accumulation of cyclin D1, PCNA and ERα. **(c)** (Data are the mean ± SD, *n* = 6) (* *p* < 0.05, ** *p* < 0.01, *** *p* < 0.001 compared to the control group; ^#^ *p* < 0.05, ^##^ *p* < 0.01, ^###^ *p* < 0.001 compared to the leptin (10 μM)-treated group)

We continued to investigate the expression of genes affected by leptin and OB3. SKOV-3 cells were treated with leptin, OB3, or their combination for 8 h. Total RNA was extracted for qPCR experiments to assess the level of expression of proliferative genes, *Cyclin-D1*, *PCNA*, *c-Myc* and *ERα*. Results shown in Fig. 2a indicated that only leptin stimulated expressions of *Cyclin-D1*, *PCNA*, and *ERα* in concentration-dependent manners (Fig. 2a). OB3 did not stimulate expression of *Cyclin-D1*, *PCNA*, or *ERα*, but rather induced a slight inhibition of their expression. Co-treatment with leptin and OB3 decreased the expression of *Cyclin D1*, *PCNA*, *c-Myc,* and *ERα* as compared to leptin (10 μM) treatment alone. The expression of *c-Myc* was induced by leptin, and the induction was inhibited by co-incubation with OB3 (Fig. 2a). Likewise, the expression of *c-Myc* was inhibited by OB3 in a concentration-dependent manner (10 μM OB3: 40.8%; 100 μM: 53.8%) (Fig. 2a).

In addition, the metastasis-related genes, MMP-2 and MMP-9 were studied. Leptin promoted expressions of MMP-2 and MMP-9 in concentration-dependent manners (Fig. 2b). There was no stimulatory effect of OB3 on MMP-2 expression (Fig. 2b). However, MMP-9 expression was inhibited by OB3 in a concentration-dependent manner (Fig. 2b). OB3 inhibited expressions of MMP-2 and MMP-9 induced by leptin. There was no significant inhibitory effect of OB3 on leptin-induced MMP-2 expression. Additionally, the expression of MMP-9 induced by leptin was inhibited by OB3 in a concentration-dependent manner (10 μM OB3: 46.3%; 100 μM: 65%) (Fig. 2B). Furthermore, in Western blot experiments, leptin significantly induced the accumulation of cyclin D1, PCNA and ERα. On the other hand, OB3 significantly inhibited cyclin D1 and PCNA. Co-treatment with leptin and OB3 revealed that ERα, cyclin D1 and PCNA were significantly diminished compared to those of leptin-treated cells (Fig. 2c).

### The inhibitory effect of the OB3 peptide on the expression of proliferative genes induced by leptin is not OB-R-dependent in ovarian cancer cells

**Fig. 3:**
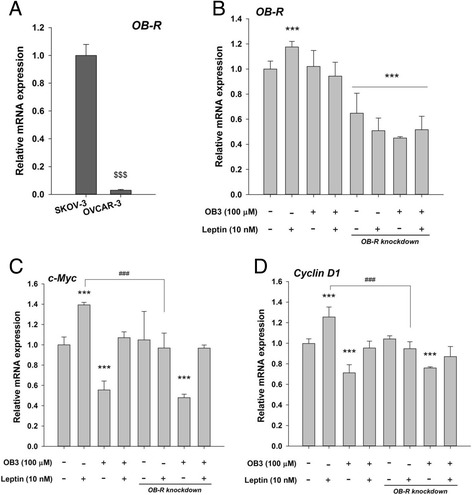
The role of the OB-R in the inhibitory effect of the OB3 peptide in ovarian cancer cells. Basal expression of *OB-R* in SKOV-3 and OVCAR-3 cells were examined by qPCR **(a)**. SKOV-3 cells were transfected with an OB-R shRNA plasmid or a scrambled plasmid. Cells were treated with either leptin (10 nM), OB3 (100 μM) or their combination for 8 h. Cells were harvested, total RNA was extracted and qPCR experiments were performed to investigate expressions of *OB-R* **(b)**, *c-Myc* **(c)**, and *Cyclin D1* **(d)**. (Data are the mean ± SD, *n* = 3) (^$$$^ *p* < 0.001 compared with SKOV-3 cells; *** *p* < 0.001 compared to the control group (scrambled plasmid transfected and non-treatment); ^###^ *p* < 0.001 compared to 10 nM leptin)

To assess the level of expression of OB-R gene in two ovarian cancer cell lines, we performed qPCR experiments with cDNA of SKOV-3 and OVCAR-3 cancer cells. The expression of *OB-R* was higher in SKOV-3, as compared with that of OVCAR-3. (Fig. 3a). To evaluate whether the OB-R was responsible for the inhibitory effect of OB3 on leptin-stimulated cell proliferative gene expression in ovarian cancer cells, shRNA was used to knockdown the expression of the OB-R in SKOV-3 cells. Treatment with leptin (10 nM) but not OB3 (100 μM) significantly stimulated expression of the OB-R (Fig. 3b). However, knockdown of the *OB-R* significantly reduced leptin-stimulated *OB-R* expression (Fig. 3b). The leptin-stimulated proliferative genes, *c-Myc* and *Cyclin D1* were inhibited by knockdown of OB-R in SKOV-3 cells (Fig. 3c, d). Furthermore, reductions in *c-Myc* and *Cyclin D1* by OB3 were still observed when OB-R was knocked down (Fig. 3c, d).

### Both leptin and OB3 activate PI3K signaling which is involved in expressions of ERα-responsive genes in ovarian cancer cells.

**Fig. 4:**
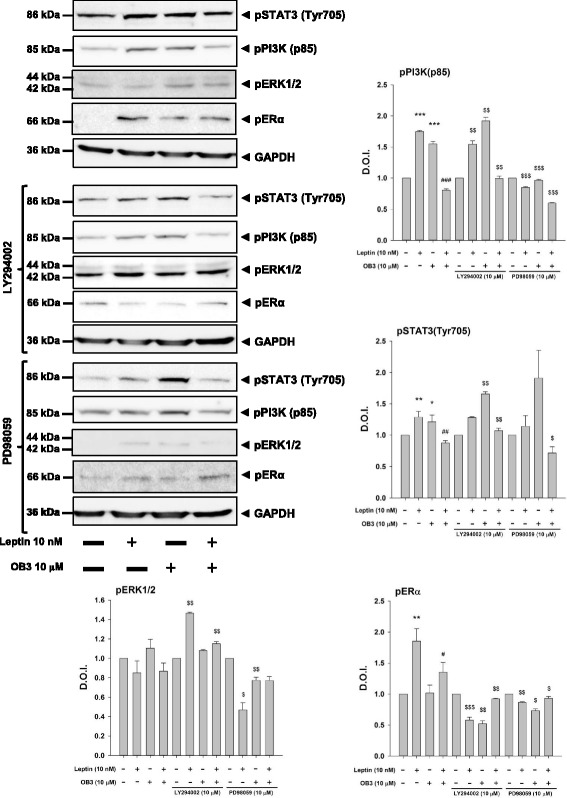
Effects of leptin and the OB3 peptide on activation of ERK1/2 and PI3K in ovarian cancer cells. SKOV-3 cells were pre-treated with LY294002 (10 μM) or PD98059 (10 μM) for 30 min and then were treated with either leptin (10 nM), OB3 (10 μM), or their combination for 3 h. Cells were harvested, and total protein was extracted. Western blot analyses were conducted to examine activation of PI3K (p85), STAT3 (Tyr705), ERK1/2, and ERα. (Data are the mean ± SD, *n* = 3) (* *p* < 0.05, ** *p* < 0.01, *** *p* < 0.001 compared to the control group (non-treatment); ^#^ *p* < 0.05, ^##^ *p* < 0.01, ^###^ *p* < 0.001 compared to 10 nM leptin; ^$^ *p* < 0.05, ^$$^ *p* < 0.01, ^$$$^ *p* < 0.001 compared to non-inhibitor treatment)

In order to investigate the signal transduction pathways involved in leptin-induced proliferation and the inhibitory effect of OB3 on leptin’s action, ovarian cancer cells were treated with 10 nM leptin, 10 μM OB3 or their combination (Fig. 4) in the presence or absence of inhibitors of ERK1/2 (PD98059) and PI3K (LY294002). Results indicated that both OB3 and leptin activated PI3K but not ERK1/2 and further induced the phosphorylation of Tyr705 of STAT3 and ERα. Activation of PI3K, STAT3, and ERα was diminished by the co-incubation of leptin and OB3 (Fig. 4) suggesting that leptin and OB3 may compete with each other for signal transduction pathways. Blockage of the signal transduction pathway by a specific inhibitor of PI3K inhibited downstream STAT3 phosphorylation in leptin-treated SKOV3 cells (Fig. 4). On the other hand, OB3-induced STAT3 Tyr705 phosphorylation was only inhibited by the ERK inhibitor, PD98059 (Fig. 4). Interestingly, activation of ERK1/2 in both leptin- and OB3-treated SKOV-3 cells was enhanced by pretreatment with the PI3K inhibitor, LY294002 (Fig. 4). The inhibitory effect of OB3 on leptin-induced activation of PI3K and STAT3 was also partially inhibited by LY294002. The activity of ERK1/2 in leptin and OB3 co-treated cells was inhibited by PD98059 and further reflected in leptin-induced phosphorylation of STAT3 Tyr-705 and ERα (Fig. 4).

**Fig. 5:**
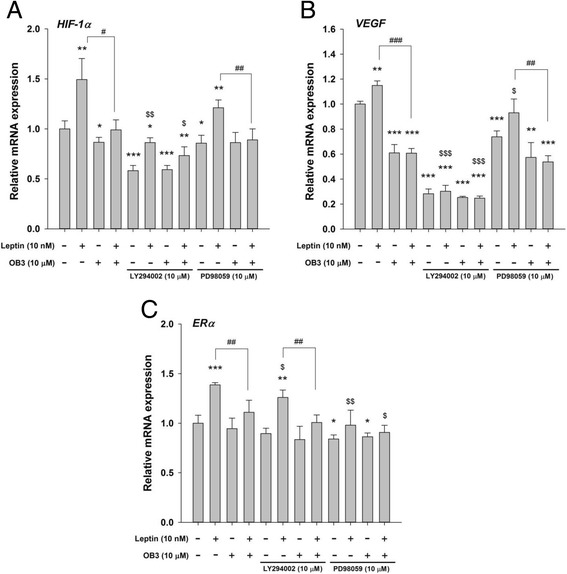
The roles of ERK and PI3K in leptin-, and OB3 peptide-, or their combination-induced expressions of ERα-responsive genes in ovarian cancer cells. SKOV-3 cells were pre-treated with LY294002 (10 μM) or PD98059 (10 μM) for 30 min and then were treated with either leptin (10 nM), OB3 (10 μM), or their combination for 8 h. Cells were harvested and total RNA was extracted. qPCR experiments were conducted to examine expressions of ERα-responsive genes: *HIF-1α* **(a)** *, VEGF* **(b)**, and *ERα* **(c)**. (Data are the mean ± SD, *n* = 6) (* *p* < 0.05, ** *p* < 0.01, *** *p* < 0.001 compared to the control group (non-treatment); ^#^ *p* < 0.05, ^##^ *p* < 0.01, ^###^ *p* < 0.001 compared to 10 nM leptin; ^$^ *p* < 0.05, ^$$^ *p* < 0.01, ^$$$^ *p* < 0.001 compared to non-inhibitor treatment)

The effects of ERK1/2 and PI3K on leptin and OB3 in terms of expressions of genes were examined. Leptin-induced expression of *HIF-1α* was reduced by treatment with PI3K inhibitor (a 42.4% drop compared to no inhibitor) **(**Fig. 5a **)**. OB3 suppressed the expression of *HIF-1α* and the inhibitory effect was slightly reduced by LY294002 compared to the untreated control with LY294002-pretreated cultures. However, LY294002 further enhanced the suppression of *HIF-1α* compared to the untreated control without added LY294002. On the other hand, although there was a significant inhibitory effect on the expression of *HIF-1α* by PD98059 in both treated and untreated controls, PD98059 did not block the expression of *HIF-1α* by leptin (Fig. 5a). In the presence of PD98059, the inhibitory effect of OB3 on leptin-induced expression of *HIF-1α* was significant compared to the same treatment without the inhibitor. Leptin induced the expression of *VEGF* (Fig. 5b). On the other hand, OB3 suppressed *VEGF* expression and significantly inhibited leptin-induced expression of *VEGF* (Fig. 5b). LY294002 blocked the effects on the expression of *VEGF* induced by leptin and OB3. It also enhanced the inhibitory effect of OB3 on leptin-induced *VEGF* expression (Fig. 5b). PD98059 inhibited the expression of *VEGF* by itself but not leptin-induced *VEGF* expression. The inhibitory effect of OB3 on leptin-induced *VEGF* expression was not affected by PD98059 (Fig. 4b). The expression of *ERα* was enhanced by leptin but not OB3, and the induction was significantly inhibited by OB3 (Fig. 5b). PD98059 and LY294002 inhibited leptin-induced *ERα* expression. On the other hand, PD98059 further enhanced the inhibitory effect of OB3 on leptin-induced *ERα* expression (Fig. 5c).

### Activation of STAT3-ERα signaling is involved in leptin-induced gene expression in ovarian cancer cells.

**Fig. 6:**
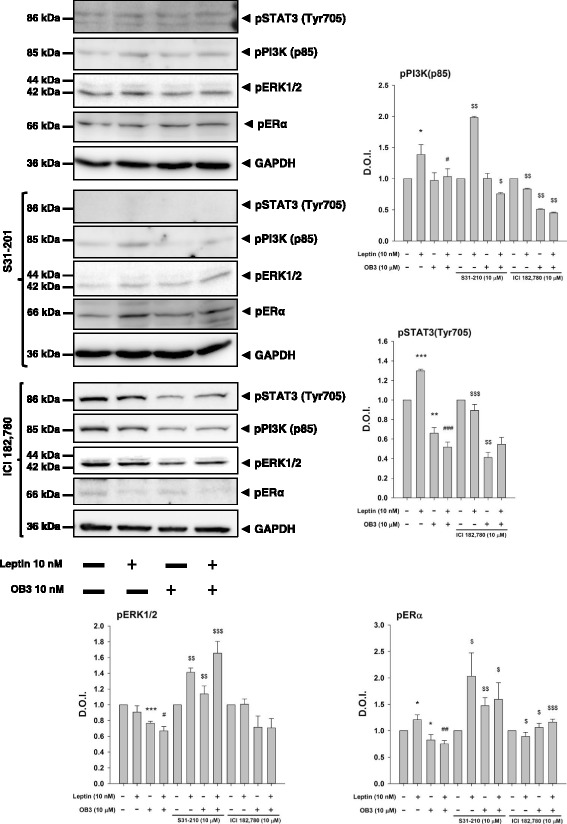
The roles of activated STAT3 and ER-α in leptin peptide, OB3, or their combination in ER-α-induced signaling activation in ovarian cancer cells SKOV-3 cells were pre-treated with S31–201 (10 μM) or ICI 182,780 (10 μM) for 30 min and then were treated with either leptin (10 nM), OB3 (10 μM), or their combination for 24 h. Cells were harvested, and total proteins were extracted. Western blot experiments were conducted to examine activation of PI3K (p85), STAT3 (Tyr705), ERK1/2, and ERα. (* *p* < 0.05, ** *p* < 0.01, *** *p* < 0.001 compared to the control group (non-treatment); ^##^ *p* < 0.01, ^###^ *p* < 0.001 compared to 10 nM leptin; ^$^ *p* < 0.05, ^$$^ *p* < 0.01, ^$$$^ *p* < 0.001 compared to non-inhibitor treatment.)

It was shown that leptin stimulates the proliferation of ovarian epithelial cancer cells partially mediated via aromatase and *ERα* [28]. Therefore, studies were conducted to examine the roles of ERα in leptin and OB3-induced biological activities in ovarian cancer SKOV-3 and OVCAR3 cells. Cells were pre-treated with either the STAT3 inhibitor, S31–201 (10 μM), or the estrogen receptor antagonist, ICI 182,780 (10 nM), and then treated with either leptin or OB3 for 1 h. Cells were harvested, and proteins were extracted for a Western blot analysis. Leptin induced ERα phosphorylation which was enhanced by S31–201 and inhibited by ICI 182,780 (Fig. 6). On the other hand, OB3 inhibited basal ERα activation which was reversed by S31–201 and ICI 182,780. However, activation of STAT3 (Tyr705) was inhibited by S31–201 and ICI 182,780 (Fig. 6).

**Fig. 7:**
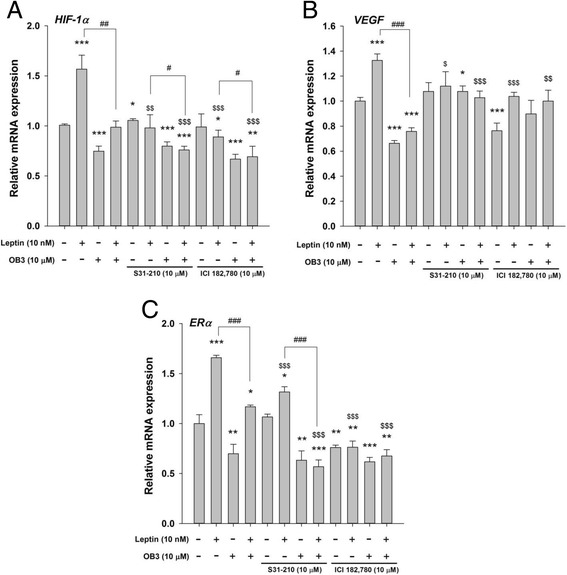
The roles of STAT3 and ERα in leptin peptide-, OB3 peptide-, or their combination-induced expressions of ERα-responsive genes in ovarian cancer cells. SKOV-3 cells were pre-treated with S31–201 (10 μM) or ICI 182,780 (10 μM) for 30 min and then were treated with either leptin (10 nM), OB3 (10 μM), or their combination for 24 h. Cells were harvested, and total RNA was extracted. qPCR experiments were conducted to examine expressions of ERα-responsive genes: *HIF-1α* **(a)** *, VEGF* **(b)**, and *ERα* **(c)**. (Data are the mean ± SD, *n* = 6) (* *p* < 0.05, ** *p* < 0.01, *** *p* < 0.001 compared to the control group (non-treatment); ^#^ *p* < 0.05, ^##^ *p* < 0.01, ^###^ *p* < 0.001 compared to 10 nM leptin; ^$^ *p* < 0.05, ^$$^ *p* < 0.01, ^$$$^ *p* < 0.001 compared to non-inhibitor treatment)

The effects of ICI and S31–201 on leptin- and OB3-regulated expression of ERα-responsive genes were examined (Fig. 7). The expression of *HIF-1α* induced by leptin was inhibited by S31–201 and ICI 182,780, indicating that activation of STAT3 and ERα was essential for leptin-induced *HIF-1α* expression. On the other hand, suppression of *HIF-1α* by OB3 was affected by neither S31–201 nor ICI, indicating that activation of ERα might not be involved in OB3-induced suppression of *HIF-1α* expression (Fig. 7a). Inhibition of leptin-induced *HIF-1α* expression by OB3 was further enhanced by S31–210 and ICI 182,780, suggesting that the inhibitory effect of OB3 on leptin-induced *HIF-1α* expression was negatively controlled by activation of STAT3 and ERα (Fig. 7a). The expression of *VEGF* by leptin was inhibited by S31–210 and ICI 182,780, suggesting that those two signals controlled the expression of leptin-induced *VEGF*. On the other hand, the suppression of *VEGF* by OB3 was reversed by S31–201 and ICI 182,780, indicating the negative role of activated STAT3 and ERα on the expression of *VEGF* by OB3 (Fig. 7b). The inhibitory effect of OB3 on leptin-induced *VEGF* expression was inhibited by S31–201 and ICI 182,780, suggesting that the inhibition was positively controlled by STAT3-ERα activation. Stimulation of ERα by leptin but not the inhibitory effect of OB3 on *ERα* expression was inhibited by S31–201 and ICI 182,780 (Fig. 7c). On the other hand, the inhibition of leptin-induced *ERα* by OB3 was inhibited by S31–201 and ICI (Fig. 7c). Those results suggested that leptin-induced ERα and the inhibitory effect of OB3 on leptin-induced ERα were positively controlled by the STAT3-ERα pathway.

### Leptin and OB3 differently affect circulating concentrations of pituitary trophic hormones in the intact mouse.

**Fig. 8:**
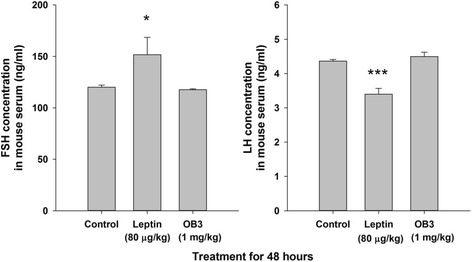
Leptin and OB3 affect circulating levels of hormones. Mice were intraperitoneally injected with 80 μg/kg leptin or 1 mg/kg OB3. At 2 days after treatment, blood samples were collected and serum samples were separated by centrifugation. Levels of FSH and LH in aliquots of serum samples were measured by competition ELISA kits and carried out according to the manufacturer’s instructions. Data are the mean ± SD, and the number of mice in each group is five. (* *p* < 0.05, *** *p* < 0.001 compared to the control group)

Leptin was shown by other investigators to increase circulating luteinizing hormone (LH) [30] and thyroid-stimulating hormone (TSH) levels [31], but not to affect FSH. Our previous studies also indicated that leptin increased circulating TSH and FSH levels [17]. We examined the effects of OB3 and leptin on levels of LH and FSH in intact mice at 2 days after leptin or OB3 administration. Results indicated that leptin (80 μg/kg) significantly increased levels of FSH but decreased those of LH (Fig. 8). OB3 (1 mg/kg), however, did not affect FSH but slightly increased LH 2 days after application. These results confirmed that leptin, but not OB3, increased FSH levels and had a positive correlation with the proliferation of cancer cells [32, 33].

## Discussion

Our results showed that the OB3 peptide did not induce cell proliferation in ovarian cancer cells. On the other hand, leptin induced cell proliferation in ovarian cancer cells which could be inhibited by co-incubation with OB3. Leptin-induced expressions of ERα-responsive genes were ERK1/2- and PI3K-dependent. In addition, activation of ERα was also observed in leptin-induced cell proliferation. The downstream signal transduction pathway, STAT3, was effective in leptin-induced proliferation.

Leptin was shown to stimulate abnormal cell growth in different types of tumors including benign and malignant ovarian epithelial cell lines [28]. In contrast, it was also shown to inhibit the cell growth of Mia-PaCa and PANC-1 pancreatic cancer cells [28] or to have no effect on the proliferation in thyroid cancer cells [17]. Unlike the parental peptide hormone, the OB3-leptin peptide did not stimulate cancer cell proliferation in cervical cancer HeLa cells or any thyroid cancer cells examined [17].

Both leptin and OB3 peptides induced phosphorylation of ERK1/2 and PI3K and phosphorylation of Ser-727 and Tyr-705 of STAT3 in human cervical cancer HeLa cells [22]. However, OB3 did not induce activation of ERK1/2, PI3K, or STAT3 in anaplastic thyroid cancer or papillary thyroid cancer cells [17]. Furthermore, OB3 reduced phosphorylation of STAT3 in follicular thyroid cancer cells. Unlike OB3, leptin induced activation of STAT3 via phosphorylation of ERK1/2 and PI3K. OB3, but not leptin, induced activation of ERK1/2 (Fig. 4). On the other hand, both leptin and OB3 activated PI3K in ovarian cancer cells (Fig. 4), which were inhibited by LY294002. Inhibition of ERK1/2 and PI3K activation further affected activation of STAT3 by OB3; however, shutdown of PI3K activation inhibited the phosphorylation of ERα by leptin and further reduced the phosphorylation of ERα by OB3 (Fig. 4). These results suggest that there are two signal transduction pathways for leptin’s action of inducing gene expression.

Aberrant activation of STAT3 was reported to promote cancer progression in many human cancers [21, 34]. Studies revealed that obesity-induced thyroid tumor growth and cancer progression are mediated by the activated phosphorylation of oncogenic JAK2 and STAT3 transcription factors [21, 34]. Both leptin and OB3 stimulated the phosphorylation of STAT3 (Fig. 5), but only leptin promoted the proliferation of ovarian cancer cells (Fig. 1). On the other hand, OB3 activated STAT3 but inhibited leptin-induced proliferation. These results suggest that activation of STAT3 might not be essential for leptin derivatives (such as OB3) to induce proliferation.

Leptin, via activated ERK1/2, increased the binding of ERα to ERα-responsive promoters [35]. Furthermore, the proliferative effect of leptin in ovarian cancer cells was inhibited by ICI 182,780 [36]. Leptin-induced cell growth and ER-α transactivation were effectively blocked by the specific STAT3 inhibitor, AG490, and to a lesser extent, by PI3K inhibition [12]. Stimulation with leptin induces STAT3 binding to ER-α.

Leptin and OB-R were demonstrated to stimulate the proliferation of ovarian cancer cells [28, 29]. The expression of *OB-R* in SKOV-3 cells was higher than that in OVCAR-3 cells (Fig. 3a). The different expression levels of *OB-R* in these two ovarian cancer cell lines were similar to those reported in studies by Choi et al. [29]. The knockdown of OB-R blocked leptin-induced expressions of *c-Myc*, *Cyclin D1* and *OB-R*, but the inhibitory effect of OB3 was not affected (Fig. 3b-d). This result suggested that the expression of proliferative genes inhibited by OB3 was not OB-R dependent.

Taken together, these results indicate that stimulation of ovarian cancer cell growth by leptin involves, at least in part, ERα transcriptional activation via STAT3 signaling pathways [12]. Studies indicated that estrogen metabolism and ERα contribute to the proliferative effect of leptin in prostate cells [15]. These conclusions suggest that leptin-induced proliferation appears to be mediated, at least in part, via aromatase and ERα [15].

The FSH stimulates cancer cell proliferation and inhibits apoptosis of ovarian cancer cells [37]. In patients with ovarian cancer, increased leptin levels are associated with a higher level of circulating FSH [38]. Treatment of mice with leptin was shown to increase circulating TSH and FSH [17]. Results presented in Fig. 8 indicate that leptin, but not OB3, increased the circulating concentration of FSH. Evidence indicates that FSH, but not LH, stimulates ovarian cancer proliferation [39]. However, results shown in Fig. 8 indicate that only leptin, but not OB3, significantly increased the concentration of FSH in serum. On the other hand, leptin reduced the LH concentration in serum. These consequences may affect the growth of ovarian cancer or other leptin-related cancers.

## Conclusions

Leptin-induced signal activation and gene expressions can be blocked by co-incubation with OB3, which is activated ERK1/2- and PI3K-dependent. Activated ERα stimulates the expression of responsive genes which may be involved in promoting cancer cell proliferation and cancer initiation. However, administration of OB3 might be able to suppress leptin-stimulated signals and the development of cancers.
